# Physiological and Biochemical Mechanisms Mediated by Allelochemical Isoliquiritigenin on the Growth of Lettuce Seedlings

**DOI:** 10.3390/plants9020245

**Published:** 2020-02-13

**Authors:** Shuang Zhang, Shi-Wei Sun, Hai-Lin Shi, Ke Zhao, Jin Wang, Yang Liu, Xiao-Hong Liu, Wei Wang

**Affiliations:** Department of Natural Medicine and Pharmacognosy, School of Pharmacy, Qingdao University, Qingdao 266071, China; qdeduzhangshuang@163.com (S.Z.); sunsw@qdu.edu.cn (S.-W.S.); shihailinjin@163.com (H.-L.S.); Qingdao_zhaoke@163.com (K.Z.); Qingdao_wangjin@163.com (J.W.); buckuper@163.com (Y.L.); liuxiaohong1043@163.com (X.-H.L.)

**Keywords:** isoliquiritigenin, phytotoxicity, *Lactuca sativa*, reactive oxygen species, cell viability, lipid peroxidation

## Abstract

Isoliquiritigenin, a natural chalcone-type flavonoid, has been recognized as an allelochemical with phytotoxicity to lettuce; however, not enough attention has been paid to the mechanisms of this secondary metabolite. In this work, we investigated the physiological and biochemical mechanisms of isoliquiritigenin on lettuce seedlings. The results show that isoliquiritigenin has a concentration-dependent inhibitory effect on radicle elongation of lettuce seedlings, but no significant impact on lettuce germination. Microscopy analyses suggest that the surface morphology of lettuce radicle tips was atrophied and the intracellular tissue structure deformed at high concentrations. Isoliquiritigenin induced the overproduction of reactive oxygen species (ROS), which led to loss of cell viability in the radicle cells. In addition, malondialdehyde (a product of lipid peroxidation) and free proline levels were found to have increased, while chlorophyll content in lettuce seedlings decreased. All these changes suggest that the primary allelopathic mechanism of isoliquiritigenin by which it inhibits radicle elongation in lettuce seedlings might be due to the overproduction of ROS, which causes oxidative damage to membrane lipids and cell death.

## 1. Introduction

Allelopathy was first introduced in 1937 by Molisch to indicate plant-to-plant interaction mediated by releasing signaling secondary metabolites through the donor plant. More than half a century later, in 1996, the definition of allelopathy was broadened by the International Allelopathy Society as ‘any process involving secondary metabolites produced by plants, microorganisms, viruses, and fungi that influence the growth and development of agricultural and biological system, including positive and negative effects’ [1]. Allelochemicals are non-nutritive substances, mostly called ‘plant secondary metabolites’ or ‘microbial decomposition products’, which can be released into the environment through natural pathways, such as leaf leaching during rain, dew, and fog; volatilization; pollen transmission; root secretion; and litter decomposition [1,2]. The allelopathic effect of allelochemicals can vary from that of inhibiting to stimulating seed germination and/or seedling growth and development of neighboring plants or other organisms, and can even be detrimental to their own species (autotoxic effect) [3]. Currently, allelochemicals are known to contribute to sustainable agricultural development and ecological protection. Many studies have shown that allelochemicals affect many aspects of plant physiological and biochemical processes, including cell division and elongation, cell micro- and ultra-structure, cell membrane permeability, antioxidant system, plant growth regulator system, protein and nucleic acid synthesis and metabolism, plant photosynthesis, water and nutrient uptake, and functions, activities of various enzymes, and so forth [1]. With the increasing number of allelochemicals characterized, the mechanism of allelochemicals on receptor plants has become a research hotspot [4].

Licorice is the dried root and rhizome of *Glycyrrhiza uralensis* Fisch., *Glycyrrhiza inflata* Bat., or *Glycyrrhiza glabra* L., which has long been used in traditional Chinese medicine (TCM) to invigorate the heart and spleen [5]. In addition to being used alone, it is one of the most frequently used TCMs, which can coordinate the characteristics of other medicines in TCM prescriptions. Besides its use as a botanical drug, licorice extracts are used for the production of food, cosmetic products, beverage, and confectionary, as well as the flavor of cigarettes due to their typical licorice flavor and distinctive sweetness [6]. With the rapidly increasing demand for raw materials, wild licorice species are endangered, and therefore *G*. *uralensis,* as a licorice resource, is widely cultivated in northwest, northern, and northeast China. However, cultivation of *G*. *uralensis* plants was hampered by replant failure, consequently leading to a reduction in yield. In earlier studies, specific allelochemicals in extracts from rhizosphere soils of *G*. *uralensis* were isolated and identified, and their phytotoxicities and autotoxicities investigated. Isoliquiritigenin was considered to be one of the key allelochemicals [7]. However, the detailed allelopathic action mechanism of isoliquiritigenin remains unknown. Clarification of the biochemical and physiological mechanism of allelopathical effects on receptor plants is essential for an in-deep understanding of this ecological phenomenon of interference among plants. Furthermore, knowledge of its mode of action might raise interest in its use in weed management. Accordingly, in this work, we investigated the phytotoxic action mechanism of isoliquiritigenin on experimental plants (lettuce seedlings in this case). To the best of our knowledge, this is the first study reporting that overproduction of ROS and lipid peroxidation may be relatively important for the phytotoxicity of isoliquiritigenin on the growth of lettuce seedlings.

## 2. Results

### 2.1. Synthesis of Isoliquiritigenin

**Scheme 1:**
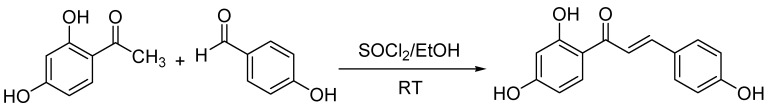
Synthesis of isoliquiritigenin.

Isoliquiritigenin was prepared via aldol condensation between 2,4-dihydroxyacetophenone and *p*-hydroxybenzaldehyde catalyzed by SOCl_2_ in ethyl alcohol (Scheme 1). Identification of the purified synthesis product by nuclear magnetic resonance (NMR) and electrospray ionization mass spectrometry (ESIMS) data provided the following results: ESIMS *m/z* 258 [M + H]^+^. ^1^H-NMR (500 MHz, acetone-*d*_6_) *δ* 8.11 (1H, d, *J* = 8.8 Hz, H-6’), 7.83 (1H, d, *J* = 15.3 Hz, H-*β*), 7.76 (1H, d, *J* = 15.3 Hz, H-*α*), 7.73 (2H, d, *J* = 8.6 Hz, H-2, 6), 6.93 (2H, d, *J* = 8.6 Hz, H-3, 5), 6.47 (1H, dd, *J* = 8.8, 2.5 Hz, H-5’), 6.37 (1H, d, *J* = 2.5 Hz, H-3’) (Figure S1). ^13^C-NMR (125 MHz, acetone-*d*_6_) *δ* 192.9 (C = O), 167.6 (C-4’), 165.6 (C-2’), 161.0 (C-4), 145.1 (C-*β*), 133.3 (C-6’), 131.8 (C-2, 6), 127.6 (C-1), 118.3 (C-*α*), 116.8 (C-3, 5), 114.5 (C-1’), 108.7 (C-5’), 103.8 (C-3’) (Figure S2). Comparing this data with the literature [8], the purified synthesis product was identified as isoliquiritigenin [1-(2,4-dihydroxyphenyl)-3-(4-hydroxyphenyl) prop-2-en-1-one].

### 2.2. Allelopathic Effects of Isoliquiritigenin on Lettuce Germination and Growth

**Figure 1:**
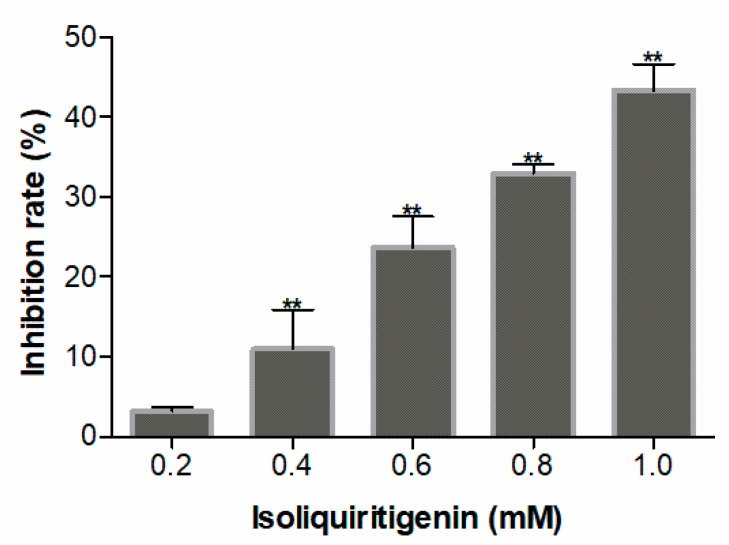
Allelopathic effect of isoliquiritigenin on radicle growth of lettuce seedlings. Values are expressed as a percentage of the mean compared to the control. Error bars are one standard deviation of the mean. *N* = 3. One asterisk * and two asterisks ** represent the significant difference between isoliquiritigenin-treated lettuce seedlings and the control at *p* < 0.05 and *p* < 0.01, respectively.

**Figure 2:**
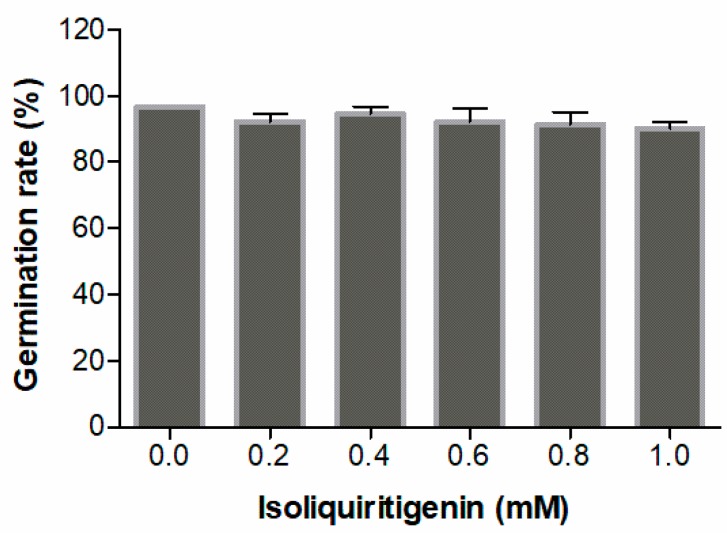
Allelopathic effect of isoliquiritigenin on the germination of lettuce seeds. Values are expressed as a percentage of the mean compared to 30 plants. Error bars are one standard deviation of the mean. *N* = 3.

Isoliquiritigenin was assayed for its effects on seed germination and seedling growth of lettuce, which is known to be a ‘standard target species’ in allelopathy research. The experimental results for seedling growth showed that isoliquiritigenin had a concentration-dependent inhibitory effect on the radicle elongation of lettuce seedlings. When the concentrations of isoliquiritigenin were higher than 0.8 mM, the radicle lengths decreased by more than 40%, as shown in Figure 1. In contrast, no differences in seed germination following any of the treatments indicated that isoliquiritigenin had no significant impact (*p* > 0.05) on lettuce germination (Figure 2).

### 2.3. Morphology Analysis

**Figure 3:**
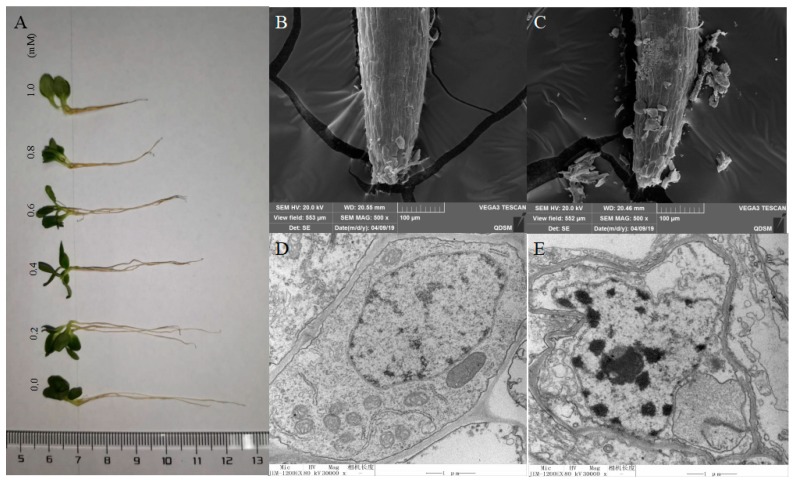
Pictures of the effect on growth length of lettuce radicles treated with isoliquiritigenin: (**A**) scanning electron microscope images of lettuce radicle surface under control (**B**) and treatment (**C**) with isoliquiritigenin (1 mM), and representative transmission electron microscope images of the ultrastructure of lettuce radicle cells under control (**D**) and treatment (**E**) with isoliquiritigenin (1 mM).

The morphology of lettuce radicle tips was photographed using a scanning electron microscope and a transmission electron microscope. Five days of exposure to isoliquiritigenin with concentrations between 0.2 to 1.0 mM inhibited radicle elongation, as shown in Figure 3A. The scanning electron microscope images showed cells in the control group arranged neatly, smooth surface, and intact cells; while, with 1.0 mM isoliquiritigenin, the surface cell gap became larger, some cells fell off, and the root tip was atrophied (Figure 3B,C). Intracellular changes were observed using a transmission electron microscope. The cells in the control group were well-formed, cell wall structure was intact, and obvious organelles were observed. After exposure to 1.0 mM isoliquiritigenin, the cell morphology changed— the cell wall was deformed and the nucleus had contracted, as compared to the control (Figure 3D,E).

### 2.4. Effect of Isoliquiritigenin on ROS Production

**Figure 4:**
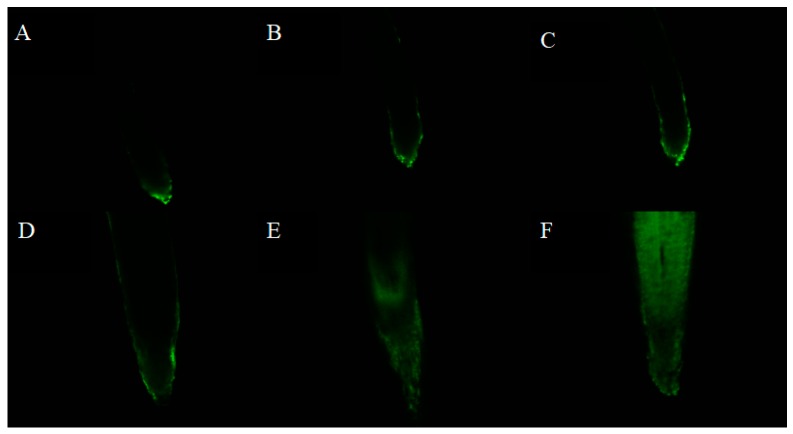
Representative pictures of ROS accumulation in lettuce radicle tips on treatment with different concentrations of isoliquiritigenin (**A**: control, **B**: 0.2 mM, **C**: 0.4 mM, **D**: 0.6 mM, **E**: 0.8 mM, **F**: 1.0 mM).

ROS production was measured in the radicle tips of lettuce after being treated with various concentrations of isoliquiritigenin by staining with 2’,7’-dichlorofluorescein diacetate (DCFH-DA) for 15 min. The results showed that the intensity of fluorescence in the tips increased markedly on treatment with increasing concentrations of isoliquiritigenin. As shown in Figure 4, slight fluorescence was induced in the radicle by the tested compound at lower concentrations (0.2 and 0.4 mM), while bright fluorescence was rapidly increased at higher concentrations (0.6, 0.8, and 1.0 mM), suggesting that isoliquiritigenin induced overproduction of ROS in lettuce radicle tips at higher concentrations.

**Figure 5:**
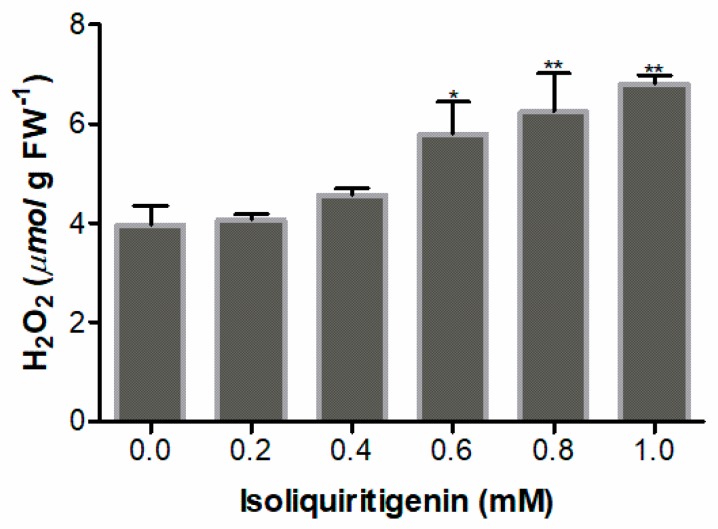
H_2_O_2_ content in lettuce radicles after treatment with different concentrations of isoliquiritigenin. Data presented are the mean ± standard deviation of results from three independent experiments. *N* = 3. One asterisk * and two asterisks ** represent the significant difference between the isoliquiritigenin-treated lettuce radicle and the control at *p* < 0.05 and *p* < 0.01, respectively.

H_2_O_2_ is a significant ROS mainly involved in the regulation of cellular metabolism. The accumulation of H_2_O_2_ was quantified to determine the influence of isoliquiritigenin on ROS accumulation in the radicle tips of lettuce. The results displayed that isoliquiritigenin induced the accumulation of H_2_O_2_ in a concentration-dependent manner. There were no differences (*p* > 0.05) in the content of H_2_O_2_ at lower concentrations (0.2 and 0.4 mM), but significant increase of 146%, 158%, and 172%, respectively, was noted when the concentration of isoliquiritigenin reached 0.6, 0.8, and 1.0 mM (Figure 5).

### 2.5. Cell Viability in Radicle Tips of Lettuce Treated with Isoliquiritigenin

**Figure 6:**
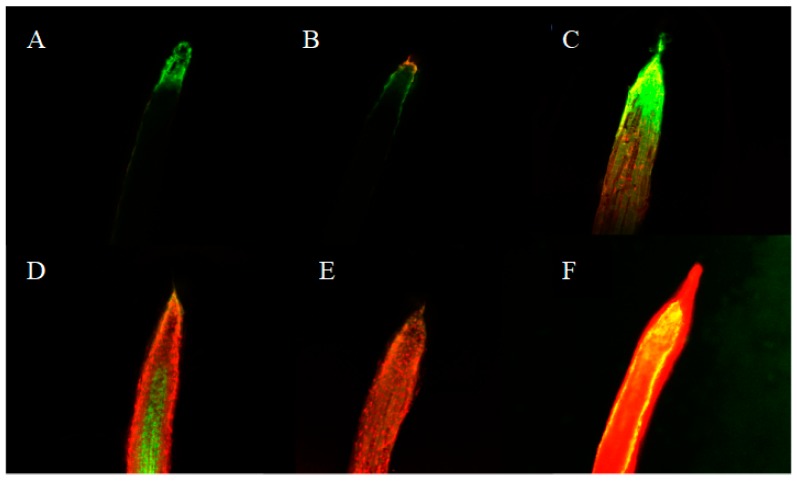
Representative pictures of cell viability in lettuce radicle tips after treatment with different concentrations of isoliquiritigenin. (**A**: control, **B**: 0.2 mM, **C**: 0.4 mM, **D**: 0.6 mM, **E**: 0.8 mM, **F**: 1.0 mM).

Cell viability is an important indicator of toxic substances that induce cell death during plant stress [9]. In this work, Evans blue uptake assays and fluorescein diacetate (FDA)/ propidium iodide (PI) double staining were used to detect the changes in cell viability of lettuce radicle after isoliquiritigenin treatment. In the double staining assay, the living cells were dyed green by FDA while the dead cells were dyed red by PI. The results showed that isoliquiritigenin induced partial loss of viability in radicle cells at low concentration (0.2, 0.4, and 0.6 mM), and most radicle cells lost viability upon treatment with isoliquiritigenin at high concentrations (0.8 and 1.0 mM), as shown in Figure 6. It can also be obviously seen that the radicle growth of lettuce was irregular (with an increased radicle diameter) when treated with more than 0.4 mM of isoliquiritigenin, compared to the control group.

**Figure 7:**
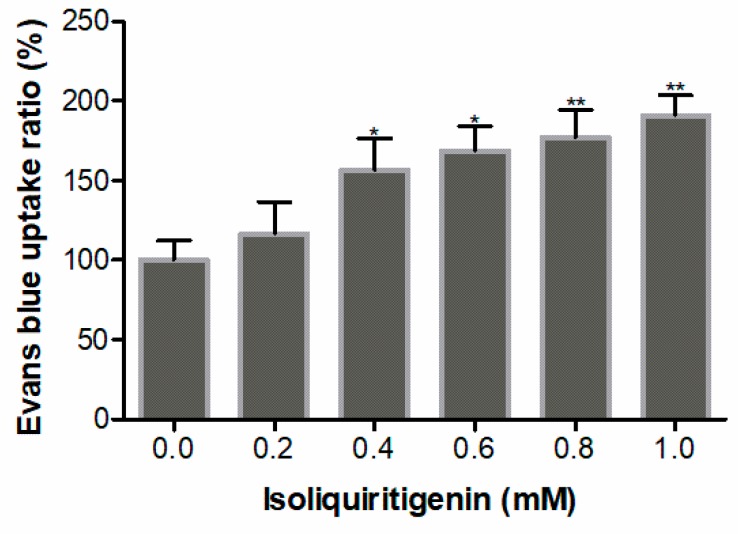
Relative Evans blue uptake in lettuce radicles after treatment with different concentrations of isoliquiritigenin. Values are expressed as a percentage of the mean compared to the control. Error bars are one standard deviation of the mean. *N* = 3. One asterisk * and two asterisks ** represent the significant difference between the isoliquiritigenin-treated lettuce radicles and the control at *p* < 0.05 and *p* < 0.01, respectively.

The cell viability of radicle tips was also determined by Evans blue uptake to quantify the rate of appearance of dead cells after isoliquiritigenin treatment. Viable cells repel harmful substances, while cells that lose vitality do not. Therefore, the relative viability of plant cells can be detected by the absorption of Evans blue by the recipient plant cells. The results show that the relative uptake of Evans blue on lettuce increased significantly by 177% and 191% after exposure to isoliquiritigenin at concentrations of 0.8 and 1.0 mM, respectively (Figure 7), which was consistent with the results of FDA/PI double staining. Therefore, these results indicate that high concentrations of isoliquiritigenin have a significant inhibitory effect on cell viability and cause cell death in lettuce radicles.

### 2.6. Effect of Isoliquiritigenin on Lipid Peroxidation

**Figure 8:**
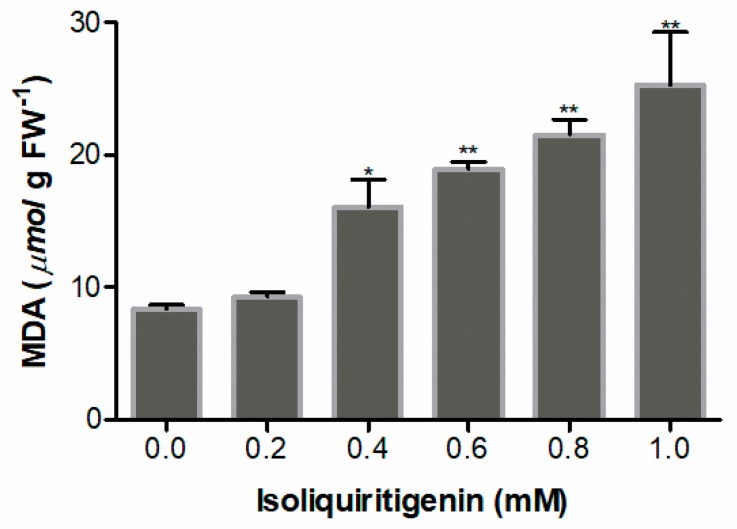
MDA content in lettuce radicles after treatment with different concentrations of isoliquiritigenin. Data presented are the mean ± standard deviation of results from three independent experiments. *N* = 3. One asterisk * and two asterisks ** represent the significant difference between isoliquiritigenin-treated lettuce radicles and the control at *p* < 0.05 and *p* < 0.01, respectively.

The level of malondialdehyde (MDA), a final product of lipid peroxidation in plants, reflects the degree of lipid peroxidation of lettuce seedlings. The results show that MDA levels in lettuce radicles increased significantly when treated with isoliquiritigenin at concentrations higher than 0.6 mM (*p* < 0.01). The highest value (303% increase) was at a concentration of 1.0 mM (Figure 8).

### 2.7. Effect of Isoliquiritigenin on Free Proline

**Figure 9:**
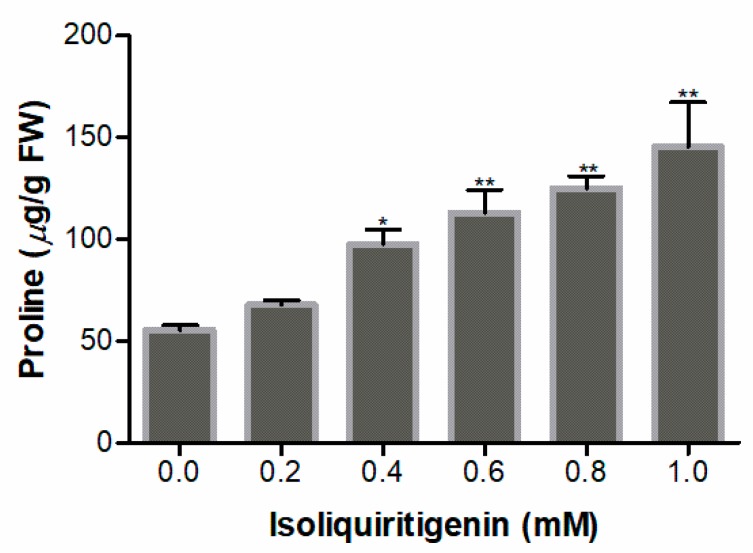
Proline content in lettuce seedlings after treatment with different concentrations of isoliquiritigenin. Data presented are the mean ± standard deviation of results from three independent experiments. *N* = 3. One asterisk * and two asterisks ** represent the significant difference between the isoliquiritigenin-treated lettuce seedlings and the control at *p* < 0.05 and *p* < 0.01, respectively.

Free proline level was measured to evaluate the stress of lettuce seedlings exposed to isoliquiritigenin. The results show that proline levels increased significantly at 0.4 mM (*p* < 0.05) of isoliquiritigenin; the highest value (263% increase) was at a concentration of 1.0 mM, compared to the control (Figure 9).

### 2.8. Effect of Isoliquiritigenin on Chlorophyll

**Figure 10:**
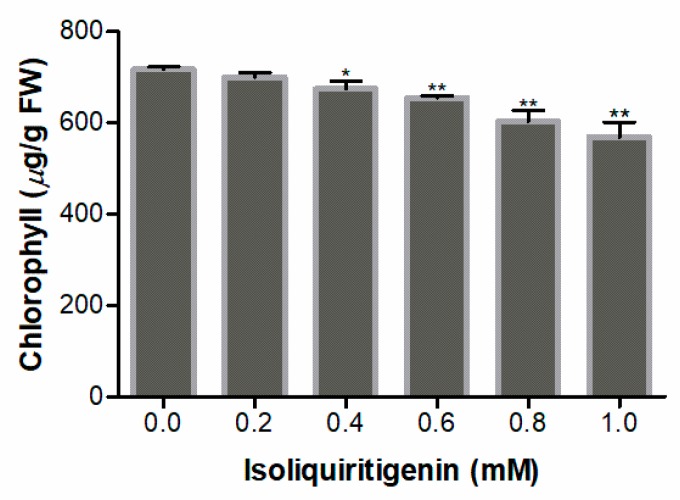
Chlorophyll content in lettuce seedlings after treatment with different concentrations of isoliquiritigenin. Data presented are the mean ± standard deviation of results from three independent experiments. *N* = 3. One asterisk * and two asterisks ** represent the significant difference between the isoliquiritigenin-treated lettuce seedlings and the control at *p* < 0.05 and *p* < 0.01, respectively.

Chlorophyll content is a representative index of plant photosynthetic ability. The reduction of chlorophyll production after treatment with isoliquiritigenin depended on the concentrations used (Figure 10). At 1.0 mM, the content of chlorophyll was 567 μg/g FW, a decrease by 21%, compared to the control.

## 3. Discussion

Isoliquiritigenin, a natural chalcone, is isolated from the roots of plants belonging to licorice. This secondary metabolite is noted for a wide range of biological activities, including anti-diabetic, antispasmodic, anti-inflammatory, antiviral, antimicrobial, anticancer, anti-oxidative, anti-angiogenic, immunomodulatory, hepatoprotective, and cardioprotective effects [10]. It was recently shown to be a key autotoxic compound in the replant problem of licorice and cause phytotoxic activity against lettuce seedlings in a concentration-dependent manner [7]. The allelopathy phenomena in plant species are responsible for natural selection. A clear understanding of their autotoxic or phytotoxic action mechanisms is crucial in investigating how to alleviate cropping obstacles, apply the observed allelopathic effects to agricultural production, and reduce pesticide input. Its remarkable phytotoxic activity on lettuce seedlings has, thus, garnered our interest.

In this study, the effects of seed germination and radicle elongation were extensively evaluated at isoliquiritigenin concentrations between 0.2 to 1.0 mM. In harmony with previous data, isoliquiritigenin exhibited inhibitory effects on the growth of lettuce seedling with dose-dependent alterations. Phytotoxicity at high concentrations of more than 100 *μ*g/mL has been observed in the literature, corresponding relating to more than 0.4 mM in our bioassay. We demonstrated that isoliquiritigenin induced more than 40% inhibition of radicle elongation in lettuce seedlings at a concentration of 0.8 mM, as many phytotoxins act as plant growth inhibitors. For example, 2-(3*H*)-benzoxazolinone, a stable decomposition product derived from the highly reactive and phototoxic but unstable plant secondary metabolite 2,4-dihydroxy-7-methoxy-2*H*-1,4-benzoxazin-3(4*H*)-one, led to 50% inhibition of lettuce radicle growth at a concentration of 0.9 mM [11]. Cyanamide, a key phytotoxic compound produced by hairy vetch, shortened tomato radicle growth by 50% at a concentration of 1.2 mM [12]. The germination of plant seeds constitutes a primary step in the growth and propagation of most plant species, and is an important parameter to evaluate allelopathic activity. Our germination assay showed that isoliquiritigenin had no significant impact (*p* > 0.05) on lettuce germination, indicating that isoliquiritigenin has an allelopathic effect on seedling growth rather than seed germination.

Cell viability is an important indicator of toxicity-induced cell death in both animals and plants. Recent studies have shown that isoliquiritigenin can inhibit the survival of tumor cells and induce the autophagy and apoptosis of cancer cells. For example, isoliquiritigenin showed significant antitumor effects on human ovarian cancer cell lines, neuroblastoma cell line, and renal carcinoma Caki cells [13,14,15,16]. In this study, the cell viability of lettuce seedlings treated with isoliquiritigenin was evaluated by FDA/PI double staining and Evans blue uptake assays. The results of the FDA/PI double staining demonstrated a considerable loss of cell viability, which was verified by the Evans blue absorption. The test compound reduced cell viability in radicle cells in a concentration-dependent manner, and induced the appearance of dead cells at high concentrations. In plants, the term programmed cell death (PCD) is widely used to describe most instances of cell death [17]. PCD is a genetically regulated physiological process of cell suicide, which is very common in the process of plant growth and development, as well as in abiotic or biotic adversity, such as cell damage after pathogen attacks [18]. It has been demonstrated that certain secondary metabolites with phytotoxic activity can induce PCD, resulting in severe alteration in plant growth. Such is the case of rosmarinic acid [19], juglone [20], hapalocyclamide [21], and cinnamic acid [22]. As a result, isoliquiritigenin is likely to cause PCD in lettuce seedlings.

PCD induction usually includes excessive ROS generation, which is utilized as a mediator of oxidative stress [23]. ROS are chemically reactive molecules containing oxygen; they are produced during the oxidative metabolism of cells. Superoxide (O^2-^) and H_2_O_2_ are the main ROS involved in the regulation of cellular metabolism [24]. Recent studies have implicated ROS in both signaling and triggering of PCD [25,26]. 2’,7’-Dichlorofluorescein is known to be relatively proportional to the intracellular level of O^2−^ [27]. In this study, the level of O^2−^ in lettuce radicles was evaluated by staining with DCFH-DA, and H_2_O_2_ contents were quantified by titanium sulfate. When isoliquiritigenin concentration reached 0.4 mM, accumulation of O^2−^ and H_2_O_2_ began, and an increase in ROS level was observed with rise in isoliquiritigenin concentration. This indicates that isoliquiritigenin effectively induces the overproduction of ROS in lettuce radicles. Other recent studies have highlighted the importance of ROS-mediated damage induced by isoliquiritigenin in A375 human melanoma cells and hepatocellular carcinoma cells [28,29]. Therefore, ROS could be a major factor causing isoliquiritigenin-induced allelopathic activity. The morphology of lettuce root tips was easy to observe by a scanning electron microscope and a transmission electron microscope. On exposure to high concentrations of isoliquiritigenin, cell morphology was found to be changed, attributing to the loss of cell viability and death. The results have suggested that isoliquiritigenin induces excessive generation of ROS, subsequently leading to membrane damage and decrease of cell viability, in addition to the initiation of PCD. Therefore, ROS might be a key factor contributing to the phytotoxicity of isoliquiritigenin. As previously mentioned, glaucocalyxin A and B and other *α*,*β*-unsaturated ketone-containing *ent*-kaurene diterpenoids have similar phytotoxic effects on plants [30]. We assume that *α*,*β*-unsaturated ketone of isoliquiritigenin may be involved in rapid Michael additions to generate a separable covalent complex with sulfhydryl of glutathione, resulting in damage to the antioxidant system of lettuce seedlings and disequilibrium between production and scavenging of ROS. However, more work needs to be done to ascertain ROS’ role as a molecular target of isoliquiritigenin on receptor plant species.

The oxidative stress produced by ROS can cause damage to cellular components, such as proteins, DNA, and lipids [31]. In this study, lipid peroxidation in cell membranes was detected by estimating MDA levels by a 2-thiobarbituric acid reaction. The increasing MDA levels indicate that isoliquiritigenin can cause oxidative damage, resulting in lipid peroxidation of membranes. Lipid peroxidation can change the permeability of cell membrane and affect their functioning, resulting in cell transparency, lesions, and fibrosis, among other damages. The accumulation of proline under environmental stress plays an important role in plant recovery from adversity due to its effect as a signaling molecule that regulates functions such as osmoregulation, detoxification of ROS, and membrane integrity [32]. The increase in proline level after isoliquiritigenin treatment suggests that the flavonoid has the ability to cause stress to the growth of lettuce seedlings. Meanwhile, the seedlings showed ROS accumulation, indicating that the chemical species may be involved in the accumulation of proline to regulate the stress induced by isoliquiritigenin. Chlorophyll level is regarded as a measure of oxidative stress [32,33]. Yan recently reported that artemisinin could reduce chlorophyll content in lettuce seedlings, and considered that chlorophyll reduction is likely linked to ROS overproduction [34]. In our study, chlorophyll content was also found to be reduced after isoliquiritigenin treatment along with an increase in ROS, indicating that ROS overproduction regulates chlorophyll content in lettuce seedlings.

## 4. Materials and Methods 

### 4.1. General

Mass spectra were acquired with a LTQ Orbitrap XL mass spectrometer (Thermo Fisher Scientific, Waltham, MA, USA). NMR spectra were obtained using a Bruker AV-500 FT-NMR spectrometer (Bruker Daltonics, Bremen, Germany). All chemical shifts are given in ppm and expressed in *δ* referring to the residual solvent signals *δ*_H_ 2.05 and *δ*_C_ 206.2, 29.8 for (CD_3_)_2_CO. Coupling constants, *J*, are in hertz. Semi-preparative HPLC was performed wirth a Megress ODS-C_18_ column (250 mm × 20 mm, i.d., 10 *μ*m, Hanbang Science and Technology, Huaian, China) on an Agilent Prepstar SD-1 pump connected to a Prostar UV-Vis detector (Agilent Technologies, Santa Clara, CA, USA). Deionized water was purified by a Milli-Q water purification system from Millopore (Bedford, MA, USA). Seeds of lettuce (*Lactuca sativa* L. var. *romana* Hort.) were purchased from Sichuan Zhongdu Seed Company (Sichuan, China). Methanol, ethanol, ethyl acetate, acetone, dimethyl sulfoxide (DMSO), sulfosalicylic acid, toluene, and hydrogen peroxide were acquired from Aibi Chemical Reagent Company (Shanghai, China). Thionyl chloride, 2’,4’-dihydroxyacetophenone, *p*-hydroxybenzaldehyde, FDA, PI, Evans blue, 2’,7’-dichlorofluorescein diacetate, titanium sulfate, and trichloroacetic acid were obtained from Aladdin Chemical Reagent Company (Shanghai, China). Sodium hypochlorite, ammonium hydroxide, and *N*,*N*-dimethylformamide were purchased from Shuangshuang Chemical Company (Yantai, China); 2-thiobarbituric was obtained from Hengyang Technology Company (Tianjin, China); glacial acetic acid, sulfuric acid, and acid ninhydrin were acquired from Beichen Founder Reagent Factory (Tianjin, China). Chromatographic grade methanol was purchased from Oceanpak (Goteborg, Sweden).

### 4.2. Synthesis of Isoliquiritigenin

In order to meet the experimental requirements of isoliquiritigenin, we synthesized isoliquiritigenin according to an improved method created by our group [35]. In brief, to a solution of 2’,4’-dihydroxyacetophenone (1 mM) and *p*-hydroxybenzaldehyde (1 mM) in ethanol (5 mL) was added thionyl chloride (0.79 mL), and then stirred at room temperature for 1 h. Towards the end of the reaction, 20 mL water was added to the mixture and extracted with ethyl acetate. Following concentration under reduced pressure, the residue was purified by reversed-phase semi-preparative HPLC with methanol/water (51/49, *v/v*) as an eluent. Eluent fraction with the desired product was collected and concentrated on a rotary evaporator to obtain isoliquiritigenin (Yield 87.2%), which was identified by NMR and ESIMS data and stored in a dark glass flask at 4 °C.

### 4.3. Evaluation of the Allelopathic Effects of Isoliquiritigenin on Lettuce Growth

The inhibitory activity of isoliquiritigenin on lettuce was evaluated according to a method described in the literature, with some modifications [36]. The seeds were surface sterilized by immersion in 10% (*v/v*) sodium hypochlorite solution for 10 min, washed three times with autoclaved deionized water, and then dried on a clean bench. A total of 30 seeds were placed on two layers of filter paper in Petri dishes 9 cm in diameter. Isoliquiritigenin was first dissolved in DMSO and then diluted with distilled water to the desired concentrations (0.2, 0.4, 0.6, 0.8, and 1.0 mM). An equal volume of DMSO was added to the distilled water as control; the final percentage of DMSO in water was less than 1%. The aliquots (6 mL) of stock solutions were added to each Petri dish. The treated seeds were incubated in a constant temperature humidity chamber at 20 ± 1 °C with 12 h/12 h dark/light photoperiod for 48 h. The number of germinations was observed every 8 h (radicle length of 1 mm).

For the growth test, the germinated seedlings (30 seedlings with radicle lengths of approximately 1 mm) were placed into the Petri dish. After incubation in a constant-temperature humidity chamber at 20 °C and a 12 h/12 h dark/light photoperiod for 5 days, radicle length was measured.

### 4.4. Determination of Cell Viability

The cell viability of lettuce was evaluated using a confocal fluorescence microscope with double staining method, as per guidelines in the literature [37]. Lettuce radicle (0.5 cm length) was cut and stained in a mixture of 12.5 μg/mL FDA and 5 μg/mL PI solutions for 10 min in the dark. The root tips were then rinsed thrice with distilled water, and observed using a confocal fluorescence microscope (Nikon A1R MP, excitation wavelength 488 nm, 561 nm and emission wavelength 520 nm, Tokyo, Japan).

The viability of radicle tips was also investigated by using an Evans blue staining method according to a procedure prescribed by Sunohara et al., with minor modifications [38]. About 0.5 g lettuce radicles (0.5 cm length) were cut and stained in a 0.25% (*w/v*) aqueous solution of Evans blue at room temperature for 1 h. The root tips were washed with distilled water for 30 min to remove surface dye. The dye was then extracted with 5 mL *N*,*N*-dimethylformamide at 25 °C for 12 h. The extraction was measured at 600 nm with an ultraviolet-visible spectrophotometer (Shimadzu, UV 2600, Kyoto, Japan).

### 4.5. Determination of O^2-^

O^2-^ level was measured by using DCFH-DA according to Xin et al. [27]. Lettuce radicles (0.5 cm in length) were cut and dyed with DCFH-DA (20 μM, 1% DMSO) for 15 min in the dark. Then, the dye residues were washed with distilled water, and the processed root tips placed on a glass slide and viewed using a confocal fluorescence microscope (Nikon A1R MP, excitation wavelength 488 nm and emission wavelength 520 nm).

### 4.6. Determination of Lipid Peroxidation

MDA content, a quantitative index of lipid peroxidation in cells, was determined by 2-thiobarbituric acid reaction, as described by Heath and Packer [39]. Lettuce radicles (0.5 g) were homogenized in 4 mL of 10% trichloroacetic acid, followed by centrifugation at 30,000 g for 10 min. Then, 1 mL of supernatant was added to 2 mL 0.6% 2-thiobarbituric acid. The mixture was heated using boiling water for 30 min, and then transferred to an ice bath to terminate the reaction. After centrifugation at 10,000 g for 10 min, absorbance of the supernatant was measured at 450, 532 and 600 nm, respectively, using an ultraviolet-visible spectrophotometer (Shimadzu, UV 2600). MDA concentration was calculated using an empirical formula prescribed by Hodges et al. [40].

### 4.7. Determination of Free Proline

Free proline, a quantitative index that reflects the resistance of plants, was determined by a method described by Bates, with modifications [41]. After treatment, about 0.2 g of lettuce seedlings was homogenized in 5 mL sulfosalicylic acid (3%, *w/v*) using boiling water for 30 min and centrifuged at 3500 g for 5 min. The supernatant (2 mL) was mixed with acid ninhydrin (2 mL) and glacial acetic acid (4 mL). After reaction using boiling water for 1 h, the mixture was transferred to an ice bath, and toluene (4 mL) was added with gently shaking. The mixture was centrifuged at 3500 g for 5 min, and the organic phase extracted and measured at 520 nm using an ultraviolet-visible spectrophotometer (Shimadzu, UV 2600). Toluene was used as a blank control. Proline concentration was obtained from a standard curve constructed with pure proline.

### 4.8. Determination of H_2_O_2_

The content of endogenous H_2_O_2_ in roots of lettuce was measured according to the literature [42]. About 1.0 g of lettuce radicle was added to acetone (8 mL) and centrifuged at 30,000 g for 10 min. To the supernatant (1 mL) was added 5% titanium sulfate (2 mL) and ammonium hydroxide (2 mL), and then centrifuged at 5000 g for 10 min. The supernatant was discarded and the precipitate completely dissolved by sulfuric acid (2 M, 5 mL). The final solution was measured at 415 nm using an ultraviolet-visible spectrophotometer (Shimadzu, UV 2600), and H_2_O_2_ contents were read from a standard curve [43].

### 4.9. Determination of Chlorophyll

After treatment, about 0.5 g of lettuce seedlings was homogenized in aqueous acetone (80%, 8 mL) and then centrifuged at 5000 g for 5 min. Absorbance of the supernatant at 645 and 663 nm was recorded using an ultraviolet-visible spectrophotometer (Shimadzu, UV 2600); then, chlorophyll a and b content was calculated according to Wellbum [44].

### 4.10. Morphological Examination

The scanning electron microscope and transmission electron microscope samples were processed as previously described, with minor modifications [45,46]. Radicle tips (0.2–0.5 cm) excised from lettuce seedlings were fixed for 4 h at 4 °C in 2.5% glutaraldehyde solution with 0.1 M phosphate buffer; the solution was discarded by washing with 0.1 M phosphate buffer three times. Then, 1.5% potassium permanganate solution was added and left to fix for 3 h. The fixed seedlings were washed in 0.1 M phosphate buffer and dehydrated through a graded series of aqueous ethanol. The selected dehydrated seedlings were rinsed with isoamyl acetate solution and dried at the critical point for examination by a scanning electron microscope (JSM-840, JEOL, Tokyo, Japan). In addition, the remaining dehydrated seedlings were rinsed with acetone and fixed in Embed 812. Ultra-thin sections (70 nm) were cut with an ultramicrotome and stained with 3% uranyl acetate in ethanol for examination by a transmission electron microscope (JEM 1200, JEOL, Tokyo, Japan).

### 4.11. Statistical Analysis

All of the bioassays were conducted in a randomized block design with at least three replicates for each treatment. The results are represented as the mean ± standard deviation. Statistical analyses were performed using a one-way analysis of variance, followed by Fisher’s least significant difference test using SPSS version 19.0 (SPSS Inc., Chicago, IL, U.S.A.) to mark variances between the treatments and the control.

## 5. Conclusions

The allelopathic inhibition of isoliquiritigenin was associated with oxidative stress stimulated by the flavanoid. Isoliquiritigenin was able to induce ROS overproduction, leading to oxidative damage, such as lipid peroxidation, and subsequent reduction of cell vitality and seedling growth inhibition. Additionally, increase in proline content and decrease in chlorophyll content were also influenced by isoliquiritigenin. Therefore, ROS may act as the primary factor in isoliquiritigenin-induced growth inhibition on lettuce seedlings.

## Supplementary Materials

The following are available online at https://www.mdpi.com/2223-7747/9/2/245/s1, Figure S1: ^1^H NMR spectrum of isoliquiritigenin, Figure S2: ^13^C NMR spectrum of isoliquiritigenin.
