# Supplementary material for: Physiological and Biochemical Mechanisms Mediated by Allelochemical Isoliquiritigenin on the Growth of Lettuce Seedlings
Source: Plants (Basel). 2020 Feb 13;9(2):245. doi: 10.3390/plants9020245 (PMC7076364; doi:10.3390/plants9020245)
Supplement: Supplementary file 1 [file plants-09-00245-s001.pdf]

Supplementary Materials

Physiological and Biochemical Mechanisms Mediated by Allelochemical Isoliquiritigenin on the Growth of Lettuce Seedlings

Shuang Zhang†, Shi-Wei Sun†, Hai-Lin Shi, Ke Zhao, Jin Wang, Yang Liu, Xiao-Hong Liu and Wei Wang*

Department of Natural Medicine and Pharmacognosy, School of Pharmacy, Qingdao University, Qingdao 266071, China; qdeduzhangshuang@163.com (S.Z.); sunsw@qdu.edu.cn (S.-W.S.); shihailinjin@163.com (H.-L.S.); Qingdao_zhaoke@163.com (K.Z.); Qingdao_wangjin@163.com (J.W.); buckuper@163.com (Y.L.); liuxiaohong1043@163.com (X.-H.L.)

* Correspondence: w.w.wangwei@263.net; Tel./Fax: +86-532-8699-1172 (W.W.)

† These authors contribute equally to this work and joint first authors.

Supporting information

Figure S-1. ^{1}H NMR spectrum of isoliquiritigenin.
Figure S-2. ^{13}C NMR spectrum of isoliquiritigenin.

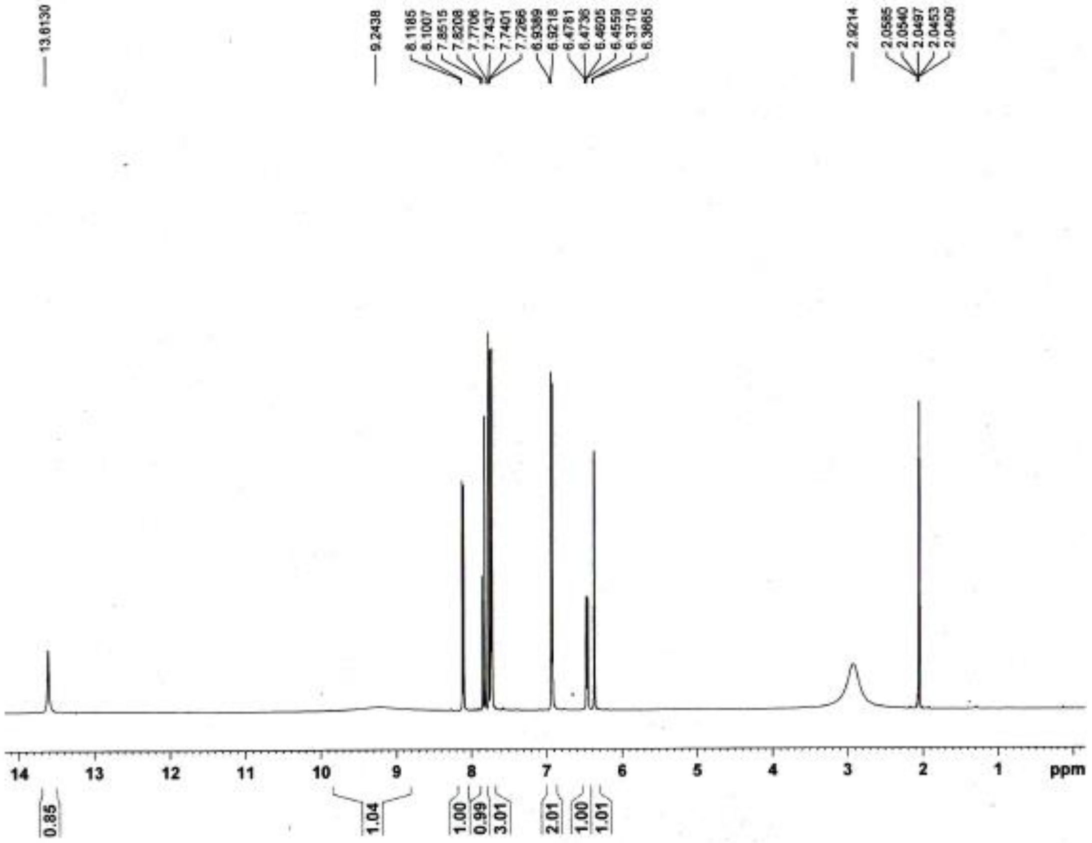

Figure S-1. ^{1}H NMR spectrum of isoliquiritigenin.

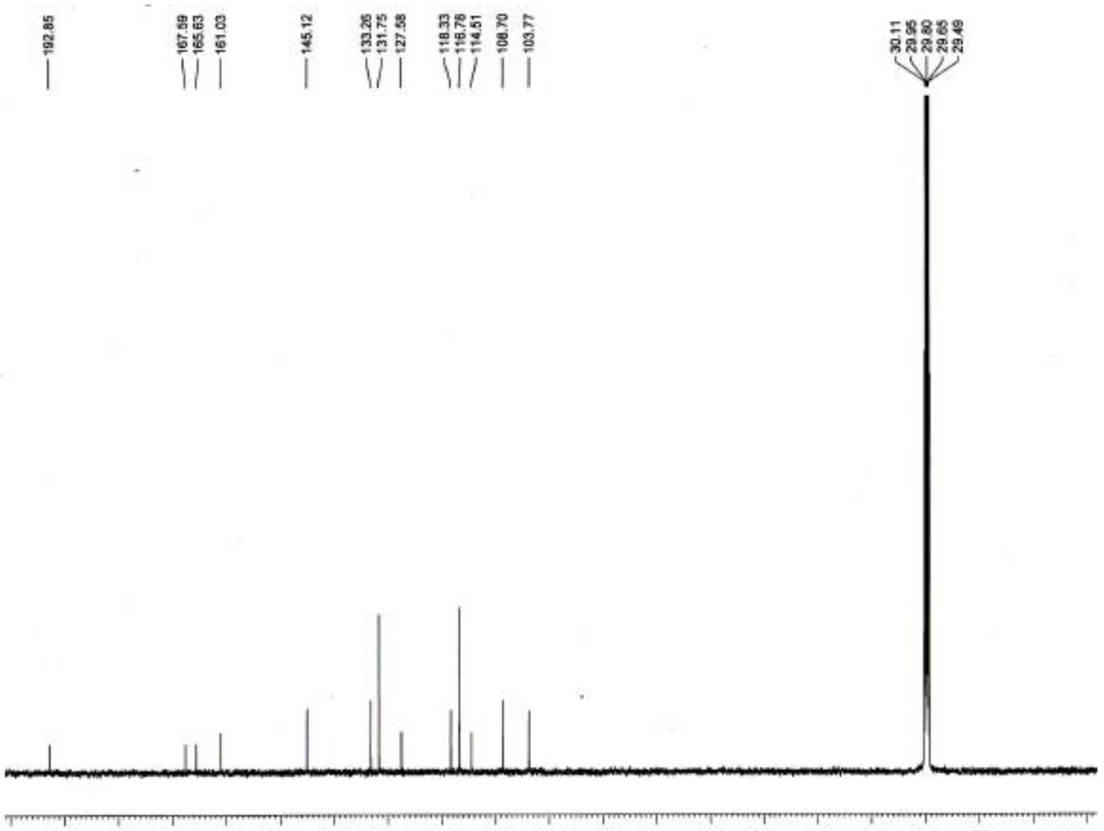

Figure S-2. ^{13}C NMR spectrum of isoliquiritigenin.
